# Molecular Imaging in Parkinsonian Disorders—What’s New and Hot?

**DOI:** 10.3390/brainsci12091146

**Published:** 2022-08-27

**Authors:** Stéphane Prange, Hendrik Theis, Magdalena Banwinkler, Thilo van Eimeren

**Affiliations:** 1 Multimodal Neuroimaging Group, Department of Nuclear Medicine, Faculty of Medicine, University Hospital of Cologne, University of Cologne, 50937 Cologne, Germany; 2 Institut des Sciences Cognitives Marc Jeannerod, CNRS, UMR 5229, Université de Lyon, 69675 Bron, France; 3 Department of Neurology, Faculty of Medicine, University Hospital of Cologne, University of Cologne, 50937 Cologne, Germany

**Keywords:** PET, SPECT, Parkinson’s disease, atypical parkinsonism, neurodegeneration, monoaminergic neurotransmission, inflammation, synucleinopathy, tauopathy, nuclear medicine

## Abstract

**Highlights:**

- Imaging of the dopamine transporter (DAT) (in particular using 11C- or 18F-PE2I) most reliably and precisely measures neurodegeneration of the dopaminergic system in parkinsonian disorders and its progression in longitudinal studies.
- Combining DAT imaging and iron- and neuromelanin-sensitive MR imaging in vivo unveiled the sequential progression of neurodegeneration along the sensorimotor, associative, and limbic territories in the striatum followed by the substantia nigra in Parkinson’s disease.
- DAT imaging is the most accurate marker for prodromal PD and predicts phenoconversion to clinical motor symptoms in patients with REM-sleep behavior disorders (RBD).
- Dopaminergic imaging can be used to investigate motor reserve in PD patients.
- Peripheral molecular imaging shows that autonomous denervation of the heart and gut in prodromal and de novo PD with RBD precedes striatal dopaminergic loss, thereby supporting peripheral starting points in those patients.
- Serotonergic denervation in the subcortical and cortical limbic regions is associated with apathy, depression, and anxiety in PD.
- Noradrenergic terminals loss plays a critical role in RBD and was shown to exceed locus coeruleus pathology in PD using 11C-MeNER PET.
- Cholinergic imaging is now complemented by the new PET tracer 18F-FEOBV, which binds to presynaptic transporters, particularly in subcortical structures.
- Dual-phase dynamic PET imaging may enable to inform both metabolic and molecular disease-related patterns with a single injection, including tracers for DAT or tau.
- Using 11C-UCB-J, prominent loss of synaptic density in the substantia nigra and brainstem was demonstrated in PD, even in the early stage, extending to the cortex in DLB, as well as PSP and CBD.
- Tau imaging using second-generation tracers with increased specificity greatly improved the diagnosis of tau aggregation in PSP and CBD.
- Despite recent advances and promising results in MSA patients using 18F-ACI12589 PET, no tracer is readily available to image α-synuclein aggregates in vivo in parkinsonian disorders.
- Computational modeling of tracer affinity in silico, coupled with cryogenic electron microscopy will improve efficiency and accelerate the development of novel tracers.

**Abstract:**

Neurodegenerative parkinsonian disorders are characterized by a great diversity of clinical symptoms and underlying neuropathology, yet differential diagnosis during lifetime remains probabilistic. Molecular imaging is a powerful method to detect pathological changes in vivo on a cellular and molecular level with high specificity. Thereby, molecular imaging enables to investigate functional changes and pathological hallmarks in neurodegenerative disorders, thus allowing to better differentiate between different forms of degenerative parkinsonism, improve the accuracy of the clinical diagnosis and disentangle the pathophysiology of disease-related symptoms. The past decade led to significant progress in the field of molecular imaging, including the development of multiple new and promising radioactive tracers for single photon emission computed tomography (SPECT) and positron emission tomography (PET) as well as novel analytical methods. Here, we review the most recent advances in molecular imaging for the diagnosis, prognosis, and mechanistic understanding of parkinsonian disorders. First, advances in imaging of neurotransmission abnormalities, metabolism, synaptic density, inflammation, and pathological protein aggregation are reviewed, highlighting our renewed understanding regarding the multiplicity of neurodegenerative processes involved in parkinsonian disorders. Consequently, we review the role of molecular imaging in the context of disease-modifying interventions to follow neurodegeneration, ensure stratification, and target engagement in clinical trials.

## 1. Introduction

Parkinson’s disease (PD) and related parkinsonian disorders are the most common neurodegenerative diseases, clinically characterized by distinctive motor symptoms and multisystem disorders. However, heterogeneity of clinical symptoms and pathology is the rule within the spectrum of neurodegenerative parkinsonian disorders. To date, post-mortem analysis enables to differentiate α-synucleinopathies, including PD, dementia with Lewy bodies (DLB), and multiple system atrophy (MSA), from tauopathies, including progressive supranuclear palsy (PSP) and cortico-basal degeneration (CBD), whereas differential diagnosis during lifetime remains probabilistic. However, molecular imaging using radioactive tracers for single photon emission computed tomography (SPECT) and positron emission tomography (PET) now enables to detect pathological changes in vivo on a cellular or molecular level with high specificity and sensitivity, related to metabolism, inflammation, transporter/receptor availability, and protein aggregates. From the clinician’s point of view, nuclear molecular imaging offers not only the possibility to detect the degeneration of the dopaminergic system but can also differentiate between the different forms of degenerative parkinsonism based on imaging of brain metabolism and neurodegeneration [1]. From the researcher’s point of view, nuclear molecular imaging can help to disentangle the complexity of the underlying neuropathology, e.g., the spread of pathogenic proteins, neuroinflammation, and imaging of non-motor symptoms [2].

**Figure 1:**
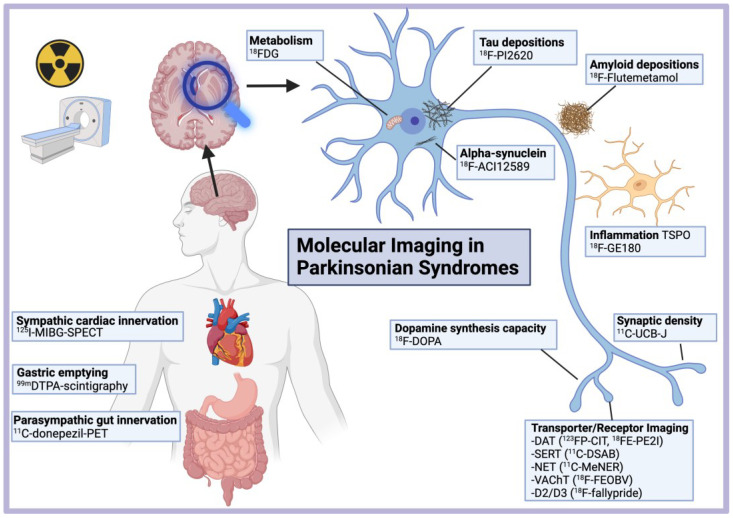
Molecular imaging in parkinsonian disorders. Created with Biorender.com (accessed on 15 March 2022).

In this review, we demonstrate how recent advances in molecular imaging using novel PET and SPECT tracers and new analytical methods are useful for the diagnosis, prognosis, and mechanistic understanding of parkinsonian disorders (PD, DLB, MSA, PSP, CBD). First, we review the advances in imaging of the neurotransmission abnormalities in PD and related disorders, involving the dopaminergic, serotonergic, noradrenergic, and cholinergic systems in the disease and the prodromal state. Second, major advances in metabolic, synaptic density, and inflammation imaging are reviewed, providing new insights into neurodegeneration and its progression across parkinsonian disorders. Third, we present the development of second-generation tau tracers and those targeting α-synuclein to image pathological protein aggregates. Finally, we review new PET tracers for promising therapeutic targets and the overall role of molecular imaging in the design and evaluation of disease-modifying interventions in parkinsonian disorders. Figure 1 gives a schematic overview of nuclear medicine and molecular imaging in parkinsonian disorders. 

## 2. Materials and Methods

We conducted a systematic literature search using PubMed and the following MeSH terms, yielding 855 occurrences between January 2019 and March 2022: ‘Parkinson’, ‘parkinsonian’, ’lewy body’, ’multiple system atrophy’, ’corticobasal’, ’progressive supranuclear palsy’ combined with ‘single photon’ and ‘positron’ for nuclear molecular imaging studies. For the part on tau imaging, we used the following MeSH terms yielding 101 results: ‘CBS’, ‘corticobasal’, ‘PSP’, ‘4R’, ‘four’ combined with ‘PET’ and ‘tau’.

## 3. Neurotransmitter Imaging

### 3.1. Dopaminergic Imaging

#### 3.1.1. Imaging the Dopaminergic System

Dysfunction of the dopaminergic system is central to the motor and nonmotor expression of parkinsonian disorders and predates the onset of overt clinical symptoms. As such, multiple investigations of the presynaptic compartment, using specific tracers for the dopamine transporter (DAT), vesicular monoamine transporter 2 (VMAT2), and aromatic-amino-acid decarboxylase were performed indicating progressive dysfunction of striatal dopaminergic terminals, besides imaging of presynaptic auto-receptors and postsynaptic dopamine receptors.

**Figure 2:**
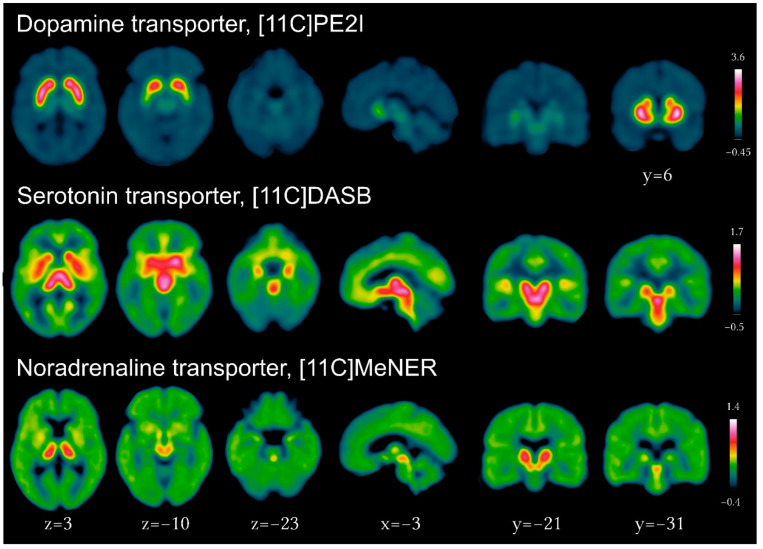
Imaging of the presynaptic dopaminergic, serotonergic and noradrenergic systems using PET tracers for the dopamine transporter ([11C]PE2I), serotonin transporter ([11C]DASB) and noradrenergic transporter ([11C]MeNER, courtesy of Christopher Doppler).

Multiple tracers have been developed for DAT using SPECT (123I-FP-CIT, 123I-beta-CIT, 99mTc-TRODAT, 123I-altropane, 123I-PE2I) and PET imaging (11C-RTI-32, 11C-CFT, 11C-methylphenidate, 11C-PE2I), of which 123I-FP-CIT and 99mTc-TRODAT are commercially available. Practice guidelines for acquisition and interpretation are available from the European Academy of Nuclear Medicine [3]. Validated tracers for the presynaptic dopaminergic (11C-PE2I), serotonergic (11C-DASB), and noradrenergic (11C-MeNER) transporters and their brain distribution in healthy individuals are presented in Figure 2. New to the field is the development and increasing use of fluorine-18-labeled PET tracers [3], next to 18F-DOPA. Indeed, longer radiological half-life relative to carbon-11 (110 min vs. 20 min) enables cost-effective distribution to imaging centers lacking on-site cyclotrons, besides advantages for new tracer design with improved affinity and selectivity, as in 18F-FP-CIT [4] and 18F-PR04-MZ for DAT [5] or 18F-FP-(+)-DTBZ for VMAT2 [6].

In particular, 18F-FE-PE2I represents a highly specific DAT tracer, enabling rapid static acquisitions as short as 10 min using high-resolution PET imaging, even 30 min after tracer injection (compared to 30 min scan time 3 to 6 h after injection for SPECT), without loss of diagnostic accuracy in comparison to standard 123I-FP-CIT scan [7]. Notably, DAT SPECT as opposed to PE2I PET also binds to the serotonin transporter, which, dependent on the research question, can be seen as an advantage or disadvantage. Importantly, it was recently shown that dopamine synthesis capacity, using 11C-β-LDOPA, and DAT availability, using 18F-FE-PE2I, were positively correlated in the putamen in healthy individuals, indicating regulated homeostasis of the synaptic dopamine concentration for normal functioning, whereas presynaptic D2 autoreceptors and post-synaptic D2 receptors striatal density were not determined by DAT or dopamine synthesis capacity [8].

#### 3.1.2. Dopaminergic Imaging in PD

Important advances were recently achieved to disentangle the complex relationship between striatal dopaminergic loss and nigral degeneration using specific radiotracers combined with magnetic resonance imaging (MRI) of magnetic susceptibility differences related to iron and neuromelanin. Indeed, it was demonstrated that striatal dopaminergic denervation and changes in the substantia nigra pars compacta involving abnormalities of iron metabolism and neuromelanin followed a sequential progression in that order, sharing a similar but time-shifted pattern along the sensorimotor, associative, and limbic territories [9]. This temporal delay likely explains the weak or absent cross-sectional association of striatal and nigral degeneration [10], although pathology is strongly concordant and lateralized to the most affected hemisphere [11]. Using diffusion-weighted imaging, degeneration of striatal dopaminergic terminals measured with 11C-DTBZ was negatively correlated with increased free-water in the posterior part of the substantia nigra, while both contributed to the prediction of motor impairment [12].

Importantly, joint pattern analysis of multiple tracers represents a new analytical method to identify patterns reflecting functional similarities and differences provided by each tracer, as for 11C-methylphenydate (DAT) and 11C-DTBZ (VMAT2) labeling in early PD [13], revealing a shared pattern of asymmetry, rostro-caudal gradient, and progression of pathology in the least-affected striatum, while DAT appeared relatively more preserved in the posterior putamen.

Greater loss of striatal DAT in the (least-affected) hemisphere ipsilateral to the clinically most affected body side was also demonstrated in a longitudinal PET study using 11C-PE2I, while striatal asymmetry gradually became less prominent despite the persistence of clinical asymmetry [14]. In addition, integration of DAT decline rate in the putamen using SPECT enables to impute the start of dopaminergic degeneration to around 10 years before motor onset in the Parkinson’s Progression Markers Initiative (PPMI) cohort [15]. Moreover, DAT-SPECT may be used to improve the prediction of motor progression using machine learning [16,17,18], as well as for cognitive outcomes in addition to cerebrospinal fluid (CSF) amyloid and tau [19]. Interestingly, patients with higher baseline levels of neurofilament light chain in the serum had faster DAT decline in the PPMI cohort [20]. Contrastingly, no definitive delineation can be drawn for tremor-dominant patients due to mixed findings regarding increased striatal DAT tracer uptake [21].

Furthermore, dopaminergic imaging enables to investigate the individual susceptibility to dopaminergic degeneration in PD patients, which is conceptualized as motor reserve and corresponds to the discrepancy between the expected motor impairment given the level of striatal dopaminergic degeneration and the individual severity of motor symptoms [22]. Importantly, motor reserve was shown to be greater with educational attainment and premorbid physical activity, and an increased degree of functional connectivity in a network involving the basal ganglia, inferior frontal cortex, insula, and cerebellar vermis, also associated with a slower increase in dopamine replacement therapy [22]. Notably, aerobic exercise may increase the release of endogenous dopamine in the striatum, as measured using 11C-raclopride PET for release evoked by repetitive transcranial magnetic stimulation [23].

#### 3.1.3. Dopaminergic Imaging in Genetic PD

Although the prevalence of monogenic inherited PD is low, great efforts have been recently conducted to identify non-symptomatic mutation carriers of autosomic PD variants (SNCA; LRRK2) and high-risk variants (GBA). This has enabled to gain new insight into the neurodegenerative and compensatory processes at work during the premotor period and possibly underpinned by differential dysfunction of dopamine synthesis and DAT [24].

Importantly, overall striatal DAT SPECT availability was found to be increased in GBA (mainly N370S) but not LRRK2 (mainly G2019S) non-manifesting carriers in comparison to healthy controls in the PPMI cohort [25], whereas lower DAT binding in the putamen was reported in 25 LRRK2 non-symptomatic mutation carriers using 11C-MP PET [26] and DAT SPECT [27], in line with previous findings [28]. Contrastingly, striatal dopamine synthesis capacity measured with 18F-FDOPA PET in LRRK2 [26] and heterozygous and homozygous GBA1 mutations carriers without PD [29] was similar to healthy controls. Interestingly, PD patients with GBA (N370S) mutations may exhibit a faster decline in striatal DAT availability during the premotor phase compared to patients with LRRK2 mutation or idiopathic PD using DAT SPECT in the PPMI cohort [15], while 18-FDOPA rate of change in GBA1 mutation carriers was similar to controls [29]. Moreover, a steady decline of DAT was previously reported in asymptomatic LRRK2 G2019S mutation carriers, comparable to the rate in patients converting to PD, which was determined by lower baseline striatal DAT availability [30,31]. However, both patients with GBA or LRRK2 mutations and overt PD symptoms in the PPMI cohort may also have increased DAT availability restricted to the striatum contralateral to the more affected body side in early PD [32]. Considering GBA variants in PD patients (excluding GBA mutations causing Gaucher’s disease), Greuel et al. found lower striatal 18F-FDOPA binding [33], also consistent with increased PD-related pattern expression [34], overall compatible with a more malignant disease course in both GBA variants and mutation carriers.

Interestingly, recent studies of the PPMI cohort demonstrated that the genetic risk as determined from the polygenic load of single nucleotide polymorphisms (SNPs) associated with PD has no effect on DAT availability in healthy subjects [35] and on annual change in PD patients, although several SNPs may influence DAT decline and deserve further investigation in larger cohorts [15,36]. This is consistent with large-scale genome-wide association studies showing no single variant associated with motor progression [37]. Interestingly, the binding of 11C-(+)-PHNO to the D2 receptor in the ventral striatum was found to be modulated by SNPs of the DRD2 gene resulting in alternative splicing into long, mainly postsynaptic, and short, mainly presynaptic autoreceptor, variants in a study with 20 PD patients [38], although replication is critical for genetic association studies.

#### 3.1.4. Dopaminergic Imaging in Prodromal PD

Due to the delay between the onset of dopaminergic pathology and motor symptoms in parkinsonian disorders, dopaminergic imaging represents the method of choice to detect early neurodegeneration at the premotor stage. It is now established that striatal (putaminal) DAT loss is the most reliable marker of prodromal PD [39] and predicts phenoconversion to symptomatic synucleinopathy within five years in patients with rapid eye movement sleep behavior disorder (RBD) [40,41,42,43] and hyposmia [44]. Inversely, normal dopamine imaging in patients with RBD has an important negative predictive value, although more than 70% of patients with RBD convert in the long term within 12 years, with an estimated annualized rate of 6% [45]. Multimodal imaging combining DAT SPECT with brain metabolic patterns and cardiac sympathetic denervation may further improve prediction for phenoconversion [46], as metabolic patterns become increasingly similar to PD [47].

Moreover, more than 25% of patients with RBD exhibit loss of the dorsal nigral hyperintensity using susceptibility-weighted MR imaging, associated with dopaminergic dysfunction, similar to PD patients [48]. Recently, dopaminergic deficit was also demonstrated in individuals with late-onset depression, exhibiting motor and nonmotor symptoms [49].

#### 3.1.5. Dopaminergic Imaging in Atypical Parkinsonian Disorders

Dopaminergic dysfunction is also found in patients with non-PD parkinsonian disorders, most prominent in those with α-synucleinopathy such as DLB and MSA. However, differentiating patients with PD or DLB using dopaminergic imaging is notably difficult (see below). In particular, striatal DAT availability was similar between early-stage patients with PD and DLB [50]. Importantly, metabolic imaging with 18F-FDG considerably improved diagnostic accuracy, characterized by a hypermetabolic pattern involving the bilateral posterior putamen, vermis, and somato-motor cortex [51]. Contrastingly, the decline of striatal DAT SPECT was greater in patients with MSA of the parkinsonian subtype (MSA-P) and of the cerebellar subtype (MSA-C) compared to PD patients [52]. Overall, dopaminergic denervation in patients with MSA is an important diagnostic marker, improving diagnostic confidence for up to 43% of patients with possible MSA-C based on clinical criteria [53], although metabolic imaging may be superior (see below).

Moreover, subtle differences in dopaminergic degeneration and compensatory mechanisms may exist between degenerative parkinsonian disorders. In particular, lower striatal D2R binding was found in a meta-analysis for PSP and MSA-P patients in comparison to PD patients (14.2% and 21.8%, respectively), contrasting with an initial upregulation of striatal D2-receptors in PD patients up to 4 years after the onset of motor symptoms [54]. Overall, multiple studies indicate that dopaminergic degeneration is more symmetric in degenerative parkinsonian disorders other than PD, although this finding is not decisive for individual diagnosis and loses its importance as asymmetry is gradually reduced in moderate and advanced PD [14,55]. As such, greater regional dopaminergic asymmetry likely involved the nigrostriatal pathway in PD patients, whereas asymmetry was restricted to the bilateral caudate in patients with DLB and to the pallido-subthalamic pathway in patients with PSP [56]. In particular, PSP patients exhibit greater symmetric dopaminergic denervation [55]. Interestingly, in patients with DLB, striatal dopaminergic denervation shown using 18F-FP-CIT contributed to multi-domain cognitive dysfunction [51,57] and motor impairment [51]. Moreover, using 18F-florbetapen PET, dopaminergic loss was associated with increased β-amyloid deposition in the putamen, parietal, lateral temporal, cingular, and occipital cortices, the latter mediating visuo-spatial dysfunction [57]. 

#### 3.1.6. Differential Diagnosis Using Dopaminergic Imaging and Use of Machine Learning

As such, imaging of the dopaminergic system is most sensitive to detect early striatal dopaminergic denervation useful for the differential diagnosis of parkinsonian disorders versus other degenerative conditions (AD, frontotemporal lobar degeneration, and other dementia) and non-degenerative parkinsonism (drug-induced, vascular, normal pressure hydrocephalus, psychogenic) and essential tremor. This has critical implications for accurate diagnosis and treatment in the vast majority of difficult cases [58]. Decision algorithm and practical issues have been previously reviewed [1,3,59,60]. Importantly, recent technical advances improved the reliability of semi-quantitative analysis of striatal binding, thanks to the better atlas-based delineation of the striatum and normative databases for age standardization [61,62].

Moreover, new automated classification methods are developed using machine-learning, including deep convolutional neural networks [63], robust to multi-site or multi-camera imaging characteristics [64], and support-vector-machine analysis [65], with performance similar to expert visual assessment across multiple centers [66]. Interestingly, extrastriatal DAT-SPECT signal greatly contributes to classification accuracy [67], in addition to the shape of the striatum [68], highlighting the need for explainable models to better depict salient features [69]. Increasingly, machine learning is also applied to metabolic imaging, highlighting the advantages of data dimensionality reduction methods such as scaled subprofile modeling [70] or LASSO regression [71].

#### 3.1.7. Implications of Advances in Dopaminergic Imaging

Altogether, dopaminergic imaging represents an early and highly reliable marker of degenerative parkinsonian disorders, useful in clinical routine for the differential diagnosis of PD and related disorders. DAT SPECT/PET imaging is increasingly considered in the design of clinical trials, both as an enrichment biomarker in early PD [72,73], enabling to increase the confidence of clinical diagnosis [74], and to follow the rate of degeneration to evaluate the efficacy of disease-modifying therapeutic interventions to promote neuroprotection [75]. As such, novel PET tracers such as 11C and 18F-PE2I are most useful to follow the longitudinal progression of dopaminergic degeneration.

### 3.2. Imaging of Peripheral Neurotransmitter in Parkinsonian Disorders

In addition to brain imaging, multisystem disorder in prodromal and overt parkinsonian disorders can be assessed using PET or SPECT, as for sympathetic cardiac imaging using 123I-MIBG SPECT or 18F-FDOPA PET, and parasympathetic small intestine and colonic innervation using 11C-donepezil, as performed simultaneously in patients with suspected prodromal α-synucleinopathy [76,77]. In particular, combined in vivo imaging of brain dopaminergic and peripheral autonomous abnormalities has enabled to support that PD patients with RBD may represent a different subtype characterized by peripheral onset of α-synucleinopathy, summarized as the body-first hypothesis [78]. Indeed, using combined in vivo evaluation of cardiac sympathetic innervation with 123I-metaiodobenzylguanidine (MIBG) SPECT and digestive parasympathetic innervation with the cholinergic 11C-donepezil PET tracer besides 18F-DOPA PET, Horsager et al. demonstrated that de novo PD patients with RBD and patients with idiopathic RBD exhibiting alterations of the locus coeruleus shared a similar pattern combining cardiac sympathetic and digestive parasympathetic denervation [76]. As such, this peripheral pattern likely preceded striatal dopaminergic dysfunction and may represent a ‘body-first’ trajectory of α-synuclein propagation, in comparison to primary striatal dysfunction in PD patients without RBD, compatible with an opposite ‘brain-first’ trajectory. Furthermore, the authors subsequently showed that de novo PD patients with RBD and patients with idiopathic RBD had significantly more symmetric nigrostriatal dopaminergic degeneration, possibly reflecting more symmetric α-synuclein spreading in the brainstem related to overlapping vagal innervation [79]. Interestingly, no difference in presynaptic VMAT2 and postsynaptic D2R dopaminergic function, using 11C-DTBZ and 11C-FLB-457, respectively, was found in PD patients with or without probable RBD, with the extrastriatal decline of 11C-FLB-457 binding in the temporal cortex, involving the uncus parahippocampus, superior, lateral, and inferior temporal cortex, although group size was limited [80,81].

### 3.3. Serotonergic Imaging in PD and Parkinsonian Disorders

Characteristic of tracers derived from cocaine analogs of tropane (CIT, TRODAT, altropane, RTI-32, CFT) is their affinity for serotonin and noradrenaline transporters, which importantly contributes to signal in extrastriatal regions with low-density DAT, as well as to noradrenaline transporters for methylphenydate and nomifensine. 

Besides dopaminergic degeneration, prominent alterations of the serotonergic projections originating in the dorsal and median raphe nuclei are observed in degenerative parkinsonian disorders. Indeed, recent studies confirm the progressive loss of the serotonin transporter (SERT) in the brainstem in PD patients using DAT-SPECT [82], with significant dysfunction of raphe nuclei in 34% of patients 4 years after diagnosis, and possibly even earlier in SNCA A53T mutation carriers, starting in the premotor phase as measured with 11C-DASB [83]. Interestingly, raphe dysfunction is subjected to considerable variability, with similar rates in tremulous and non-tremulous patients [82]. Recent analysis of extrastriatal DAT-SPECT in the PPMI cohort indicates lower uptake in frontal, temporal, and posterior cortical regions, progressing over one year and correlated with the severity of motor symptoms, cognitive performance, and CSF α-synuclein levels [84].

Analysis of multimodal PET imaging of DAT using 11C-MP, VMAT2 using 11C-DTBZ, D2R using 11C-raclopride, and SERT using 11C-DASB revealed a pattern related to motor complications. This pattern is critically related to serotonergic denervation and associated with higher dopamine release and dopaminergic degeneration in the putamen, thus corresponding to abnormal dopamine turnover in early PD [85]. Furthermore, double-tracer PET studies of the dopamine and serotonin transporters demonstrated the prominent role of SERT dysfunction in the limbic system associated with apathy, depression, and anxiety in de novo [86] and moderate-to-advanced PD patients [87]. Moreover, longitudinal follow-up of de novo apathetic patients indicated a specific increase in serotonergic innervation in the ventral striatum and anterior cingulate cortex, associated with the reversal of apathy independently of dopamine replacement therapy [88]. Altogether, there is growing evidence that the serotonergic system might be subjected to compensatory changes in early PD, as in non-symptomatic LRRK2 mutations carriers [26]. Moreover, it was recently shown that changes in cerebral serotonin levels occur when DBS is turned off, with decreased 11C-AZ10419369 binding to 5HT1B receptors, possibly corresponding to increased serotonin levels in the temporal, limbic, and occipital cortices, dependent on regional receptor preservation [89].

In addition, patients with DLB have greater serotonergic degeneration in the thalamus [90] and hippocampus [50], likely more pronounced and involving the amygdala in patients with DLB and CSF AD biomarker profiles [91]. Furthermore, lower SERT binding of DAT SPECT was found in MSA-P and PSP in comparison to PD and MSA-C patients [92]. In addition, lower 18F-MPPF binding to 5HT1A receptors was shown in the raphe nuclei, caudate, and thalamus in patients with MSA when compared to healthy controls [93].

### 3.4. Noradrenergic Imaging in PD and RBD

Noradrenergic imaging is particularly challenging, given that current radiotracers derived from antidepressants are non-selective to the noradrenalin transporter (NET), but also bind to DAT and SERT [94]. However, new, more specific, PET tracers are developed, such as for alpha2-adrenoreceptors [95].

Using 11C-MeNER PET, Doppler et al. demonstrated that noradrenergic terminal loss exceeds cellular locus coeruleus degeneration measured using neuromelanin-sensitive MRI in PD [96], playing a critical role in RBD and disorganization of sleep microstructure [97]. Interestingly, a recent PET study using 11C-MeNER demonstrated that noradrenergic innervation is preserved in tremor-dominant PD patients, involving the locus coeruleus and thalamus [98]. Moreover, monoamine synthesis capacity in the putamen measured with 18F-FDOPA and noradrenergic dysfunction were correlated in patients with idiopathic RBD, who also had decreased 11C-MeNER binding in the primary sensorimotor cortex [99], likely reflecting early noradrenergic dysfunction in prodromal α-synucleinopathy.

In addition, cardiac sympathetic noradrenergic denervation can be assessed using 123I-MIBG SPECT and 18F-FDOPA PET. This provides information for the differential diagnosis of PD/DLB versus other parkinsonian disorders, and on the degree of autonomic dysfunction [100], which can be coupled to the assessment of brain dopamine synthesis in one session [101]. 

### 3.5. Cholinergic Imaging in PD

The development of novel cholinergic fluorine-based PET tracers, including 18F-Fluoroethoxybenzovesamicol (18F-FEOBV) binding to the vesicular acetylcholine transporter [102], has enabled rapid advances in the last five years to pinpoint the role of basal forebrain cholinergic dysfunction for falls and freezing of gait in PD [103], as well as multi-domain cognitive impairment [104], as reviewed in [105]. Indeed, in contrast with 11C-MP4A or 11C-donepezil, which indicates cholinesterase activity in both the pre- and post-synaptic compartment and synaptic cleft, but with low sensitivity in subcortical structures, 18F-FEOBV binds with high affinity to the vesicular transporter, notably in the striatum [106]. Notably, no tracer is currently available to image the neurotransmitter synthesis by the choline acetyltransferase. Most interestingly, van der Zee et al. demonstrated that PD patients without cognitive impairment had higher-than-normal binding in cerebellar, frontal, and subcortical regions possibly reflecting early compensatory cholinergic upregulation [107], although all patients had lower 18F-FEOBV in the posterior cortical region. In addition, patients with RBD exhibited increased 18F-FEOBV uptake in brainstem nuclei involved in muscle atonia, deep cerebellar nuclei, as well as in limbic territories of the thalamus, anterior cingulate, and orbitofrontal cortex [108]. Contrastingly, acetylcholinesterase levels assessed with 11C-donepezil PET were reduced in the superior temporal, occipital, cingulate, and dorsolateral prefrontal cortices [109].

In comparison, patients with DLB had a prominent reduction in 18F-FEOBV in the lateral geniculate nuclei, pulvinar, optic radiation, thalamus, fimbria, fornix, dedicated to visual attention and spatial navigation, but also the anterior and mid-cingulate cortex and insula in comparison to age-matched healthy controls [110], most likely involved in disabling and fluctuating symptoms in DLB.

Altogether, in vivo imaging of neurotransmitter abnormalities in the dopaminergic, serotonergic, noradrenergic, cholinergic, and autonomous systems has benefited from multiple advances in recent years, useful for the early diagnosis and the pathophysiology of nonmotor symptoms in parkinsonian disorders. In particular, PET tracers for DAT as 11C-PE2I and 18F-PE2I have demonstrated their utility to follow dopaminergic degeneration along time, which can be evaluated in neuroprotection trials. Furthermore, novel radiotracers are currently under development, such as dopaminergic D3 selective radiotracers [111], higher-affinity DAT tracers useful for extrastriatal DAT imaging such as 18F-PR04.MZ [5,112], but also cholinergic imaging such as for 18F-FDEX, a non-subtype selective tracer of muscarinic receptors evaluated in human [113,114] and A2A (adenosine) receptors with 18F-TOZ1 [115]. Most interesting is the development of radiotracers binding to LRRK2, including 18F-FMN3PA, 18F-FMN3PU, and 11C-GNE1023 [116,117]. PET imaging of the opioid system, using radiotracers selective to receptor subtypes [118], and of endocannabinoids, as with 11C-CURB binding to the endocannabinoid enzyme fatty acid amide hydrolase [119], may also prove critical for the understanding of non-motor symptoms in PD and related disorders.

## 4. Imaging of Metabolism, Synaptic Density and Inflammation

Metabolic imaging using 18F-fluorodeoxyglucose (FDG) PET is central for the distinction between PD and other neurodegenerative parkinsonian disorders. Indeed, metabolic imaging provides specific and sensitive markers of neurodegeneration in DLB, MSA, and PSP, as a result of dysfunction of neural glucose uptake determined by synaptic activity. Furthermore, major advances were achieved in recent years using multivariate models for metabolic imaging, demonstrating characteristic, disease-related, patterns of metabolic activity, consisting in a reproducible and specific relative regional increase or decrease in glucose uptake. As such, a specific PD-related disease pattern (PDRP) was first demonstrated in PD and recently extended to other parkinsonian conditions and to specific symptoms.

### 4.1. Metabolic Imaging in Parkinsonian Disorders

The cingulate island sign, defined as the relative sparing of posterior cingulate cortex metabolism engulfed in widespread posterior cortical hypometabolism in the adjacent precuneus and cuneus, represents one of the one most visually striking markers of DLB, contrasting with early cingular hypometabolism in AD [120]. Interestingly, relative posterior cingular SPECT hypoperfusion was identified as a specific feature in hypothesis-free deep-learning classification using a deep convolutional neural network trained on perfusion SPECT [121]. In addition, regional hypometabolism was shown to be closely associated with core clinical features of DLB, whereas more preserved metabolism was maintained across the spectrum of clinically heterogeneous DLB patients [122].

Considering patients with MSA, widespread frontal, temporal, parietal, and limbic hypometabolism is observed, specifically involving the posterior cingulate for cognitive impairment [123]. Besides the involvement of the limbic temporal hypometabolism in lower cognitive performance [124], left insular hypometabolism independently contributed to worse survival in MSA next to orthostatic hypotension and stridor [125]. Lower metabolism is found in the cerebellum in patients with MSA-C compared to MSA-P [124]. Interestingly, latent class analysis of clinical heterogeneity in probable MSA patients newly identifies a profile characterized by prominent cerebellar, cognitive symptoms and greater cerebellar hypometabolism in a minority of patients [126]. 

Although it is still unclear which pathological mechanisms drive changes in brain metabolism, recent studies demonstrated joint regional evolution of metabolism and striatal dopaminergic and thalamic serotonergic innervation in de novo PD patients. In this context, metabolism mediated the relationship of caudate dopaminergic degeneration with bilateral posterior temporo-parietal metabolism on the one hand, and of serotonergic thalamic innervation with prefrontal and insular cortical metabolism on the other hand [127]. Furthermore, midbrain hypometabolism involving the substantia nigra pars compacta was recently evidenced in PD patients, associated with ipsilateral putaminal dopamine synthesis capacity using 18F-FDOPA [128]. In addition, brain metabolism was shown to gradually decline over the default mode network, most pronounced in PD patients with mild cognitive impairment [129]. Most interestingly, dopaminergic deficiency using DAT-SPECT was associated with increased relative glucose metabolism in the striatum and limbic system in patients with DLB [130]. Altogether, dopaminergic imaging combined with 18F-FDG PET has critical implications for diagnosis in the clinical context, changing diagnosis in 14% of patients referred for molecular imaging [131].

### 4.2. Disease-Related Patterns Using Metabolic Imaging

Whereas the resting-state network defined by spatial covariance of glucose uptake is topographically similar to the default mode network in healthy individuals [132], regions of relative hypo- or hypermetabolism reveal novel disease-specific patterns, first demonstrated in PD. Importantly, these specific patterns are highly reproducible and therefore useful for the classification of PD patients versus healthy controls, and show a gradual longitudinal increase with motor progression at the individual level [133]. As recently demonstrated using graph theoretical sparse network derivation, major nodal hubs primarily involve the low metabolic angular gyri and inferior parietal regions with strong cross-coupling acting the more metabolically active putamen and pallidum [134]. Interestingly, PDRP expression is more symmetrical and largely uncoupled from dopaminergic asymmetric dysfunction [135]. Similarly, symptom-specific patterns were also derived from 18F-FDG PET for cognitive dysfunction (PDCP) [136]. In addition, other specific disease-related patterns were recently demonstrated, providing high classification accuracy for DLB [51], MSA, and PSP variants [137], as reviewed in [133]. A close similarity of disease patterns is found for PD and DLB [51].

Most interestingly, PD patients with GBA variants had higher PDRP and PDCP expression, involving the whole network, compared to patients with idiopathic PD and to PD patients with LRRK2 mutations [34]. This is congruent with a known more malignant disease course. Contrastingly, preferential gain in connectivity was observed within the PDRP core of metabolically active regions for PD patients with LRRK2 mutations and associated with maintenance of lower disease network expression [34], compatible with the suspected more benign course. Moreover, higher PDRP expression was found in PD patients with RBD, besides lower striatal DAT availability [138], with spatial overlap of PDRP and PD-RBD-RP defined in de novo PD patients and moderately increased in RBD patients without dopaminergic deficit [139]. Moreover, it was shown that RBD-RP expression tended to decrease over time, while PDRP increased in most patients and was higher in those who converted to PD [140], consistent with recent findings [47].

**Figure 3:**
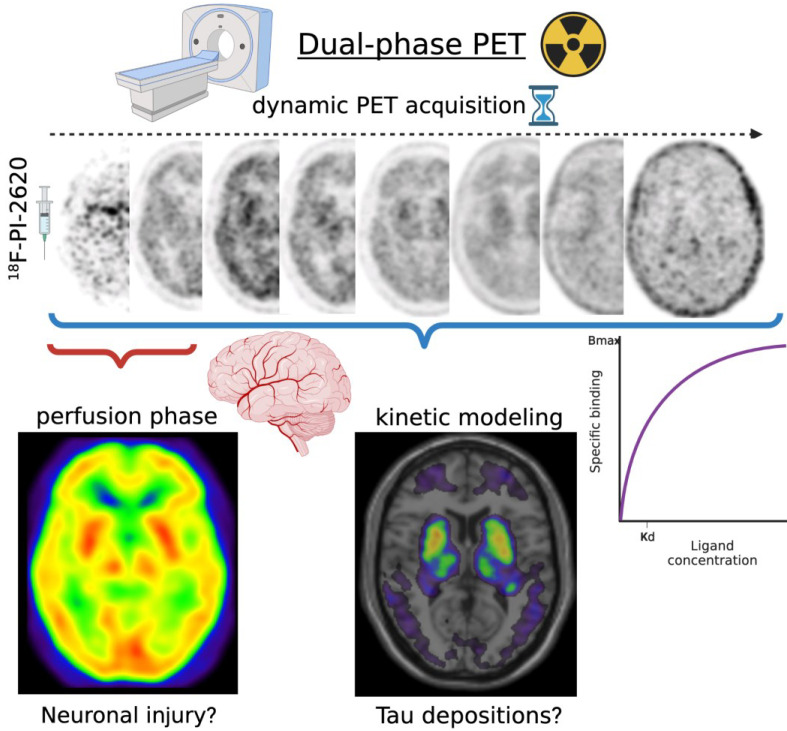
Schematic illustration of dual-phase PET imaging using 18F-PI2620 PET. Created with Biorender.com (accessed on 15 March 2022).

Therefore, disease-related expression patterns derived from metabolic imaging have now been established as particularly useful for diagnostic, possibly surpassing dopaminergic imaging [51], with multicentric and international validation, as well as an important tool to investigate neurodegenerative mechanisms from multivariate data. Great potential therefore lies in dual-phase imaging (Figure 3), using the early perfusion-like phase of PET and SPECT tracers (including those for DAT, tau, and amyloid), which is closely related to the metabolic signal, to derive the expression for disease-related patterns in addition to functional imaging of the dopaminergic system [141,142].

### 4.3. Imaging of Synaptic Density

Important advances have been achieved using 11C-UCB-J PET binding to the synaptic vesicle glycoprotein 2A (SV2A) to measure synaptic density in vivo across neurodegenerative disorders, showing consistent patterns of SV2A loss in AD and PD, partly overlapping with hypometabolism [143]. In particular, important decreased 11C-UCB-J binding was found in PD patients with bilateral disease, most pronounced in the brainstem involving the substantia nigra (−45%), red nucleus (−31%), and locus coeruleus (−17%), with post-mortem validation [144]. Lower synaptic density in the substantia nigra was confirmed in early PD patients [145] and may be more widespread even in drug-naive PD patients [146], while synaptic density correlated with DAT availability [145] and 11C-SA-4503 PET signal (binding to the sigma 1 receptor (σ1R), a chaperone protein present in the membrane of the mitochondrion-associated endoplasmic reticulum) in the caudate nucleus [146]. Interestingly, synaptic density loss is particularly prominent and widespread in patients with DLB and PD dementia in the cortex, while nigral loss is found in both non-demented and demented patients [147].

In addition, 11C-UCB-J binding was also reduced in both PSP and CBD patients in comparison to healthy controls, most pronounced in the midbrain, frontal, temporal, parietal, occipital and cingular cortices, basal ganglia, thalamus, hippocampus, insula, and amygdala [148]. It is expected that the availability of new fluorine tracers (18F-SynVesT-1 and 18F-SynVesT-2) will enable more centers to further investigate changes in synaptic density, fulfilling the need for larger cohorts.

### 4.4. Imaging of Inflammation

Imaging of inflammation relies on multiple PET tracers targeting the 18 kDa translocator protein (TSPO), highly expressed in microglial cells recruited in brain inflammation, although no distinction can be made between pro- and anti-inflammatory processes. As such, the first-generation radiotracer 11C-PK11195 was extensively used, albeit limited by low signal-to-noise ratio and high nonspecific binding. As such, widespread increased binding of 11C-PK11195 is found in PD patients, as indicated by a recent meta-analysis [149], although the number of studies remains limited, and findings are mixed. Interestingly, elevated 11C-PK11195 was found in asymptomatic LRRK2 mutation carriers, restricted to those with impaired putaminal dopamine synthesis assessed with 18F-FDOPA [150] and in GBA1 mutation carriers without PD [151]. In PSP, 11C-PK11195 binding in the brainstem (midbrain and pons), dentate nucleus, and cerebellar white matter predicted clinical progression, besides 18F-AV1451 [152].

Therefore, a variety of new PET radiotracers were developed, such as 11C-PBR28, 18F-PBR06, 11C-DPA-713, 18F-DPA-714, 18F-FEDAA1106, 11C-DAA1106, or 18F-FEPPA (see for review [153]), although ubiquitous brain expression of TSPO intrinsically limits signal-to-noise ratio and choice of reference region for modeling, besides challenging pharmacokinetics of some second-generation tracers [154].

Moreover, a single nucleotide polymorphism rs6971 of a TSPO gene modulates the binding of second-generation tracers with low, high, and mixed affinity binders [155]. As such, despite well characterization in a group of 8 high and 8 mixed affinity binders, no alteration of 11C-PBR28 is found in PD patients in comparison to healthy controls [156], confirming previous findings with 18F-FEPPA [157,158]. Using 18F-DPA714, elevated binding was found in the midbrain and putamen, but also frontal cortex in PD patients with mixed or high-affinity [159]. Interestingly, elevated 11C-PBR28 binding with hotspots in the cerebellar white matter and lentiform nucleus clearly separated patients with MSA from PD patients [160], although binding may be biased by group differences of non-displaceable binding [161].

Therefore, new, third-generation, radiotracers of TSPO are developed to avoid modulation by the rs6971 SNP. Among those, 18F-GE180, 11C-ER176, and fluorine analogs [162] are still under evaluation in inflammatory brain disease [163]. Most interestingly, a recent study found elevated 18F-GE180 binding in patients with PSP and CBS in the internal pallidum, and in the motor and supplementary motor cortex in those with CBS [164].

Furthermore, new targets are represented by imidazoline 2 for astrogliosis using 11C-BU99008 PET, with preliminary findings indicating increased uptake in the frontal, temporal, parietal, and occipital cortices in PD [165], and monoamine oxidase B expressed in reactive astrocytes using 18F-THK5351, also binding to tau and displaying longitudinal increase in patients with CBS [166], and the more selective 11C-SMBT1 PET tracer [167]. Moreover, 11C-JNJ717 PET binding to the P2X7 receptor predominantly expressed in activated microglia was evaluated in PD patients [168]. In addition, PET imaging of pro-inflammatory cells using 64Cu-TREM1-mAb for the triggering receptor expressed on myeloid cells 1 (TREM1) may represent a promising strategy [169], as for 11C-CPPC binding to the macrophage colony-stimulating factor 1 receptor expressed in microglia [170], in preclinical models.

## 5. Imaging of Protein Aggregates

### 5.1. Second Generation Tau Imaging

Tau depositions can be uncovered in vivo by tau PET, which is critical for the so-called 4R-taupathies including CBD and PSP. First-generation tau tracers such as Flortaucipir, aka 18F-AV-1451, are used to detect tau depositions in PSP or CBD and to discriminate patients from controls. Nevertheless, the PET signal did not seem to be specific to 4R-tau but was rather associated with off-target binding [171,172]. For a detailed review about tau imaging in atypical parkinsonism with first-generation tau tracers, see [171]. Therefore, new, second-generation, tau PET tracers were developed to increase specificity and reduce off-target binding [173], including 18F-PI-2620, 18F-RO-948, 18F-MK-6240, 18F-JNJ-311, 18F-JNJ-311, 18F-GTP1, and 18F-PM-PBB3. 

**Figure 4:**
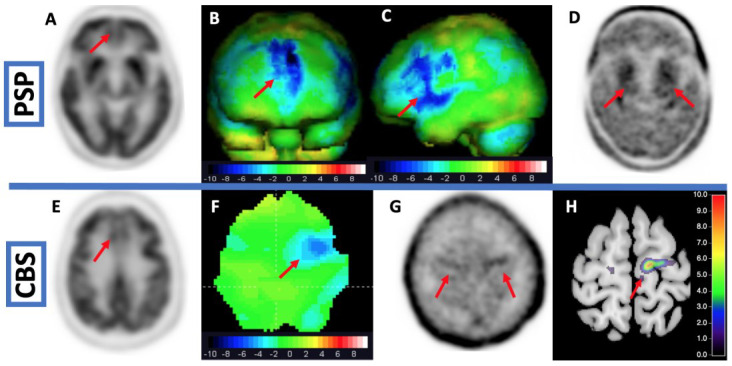
Top row: 60-year-old woman with falling, vertical gaze palsy and agrammatic language. (**A**) Native FDG-PET. (**B**) + (**C**) Z-deviation map in comparison to a healthy inhouse control group. The FDG-PET shows hypometabolism in the frontomesial and left frontolateral cortical regions, suitable but not sufficient for the diagnosis of PSP. The subsequent tau PET with 18F-PI-2620 (**D**) shows tau depositions in the basal ganglia. Diagnosis of a probable PSP could be confirmed. Bottom row: 70-year-old patient with dystonia and hyperreflexia of the right arm, and apraxia. DAT-SPECT was normal. FDG-PET revealed hypometabolism in the frontomesial cortical region and in the left precentral gyrus. (**E**) Native FDG-PET. (**F**) Z-deviation map in comparison to a healthy inhouse control group. The subsequent tau PET with PI-2620 (**G**) shows tau deposition in the bilateral precentral gyrus, accentuated on the left side. (**H**) Z-deviation map of the PI-2620 binding potential in comparison to a healthy inhouse control group. Normal DAT-SPECT and missing tau deposition in the basal ganglia contrasted with cortical tau deposition, favoring the diagnosis of CBS with underlying 3R/4R-tauopathy (AD) versus 4R tauopathy as CBD. Additional amyloid-PET (cortical amyloid deposition) or CSF biomarker (reduced α-Amyloid-1-42) may help to confirm diagnosis.

In a cross-sectional study using 18F-PI-2620 [174], patients with PSP were compared to patients with PD and MSA and healthy controls. Additionally, the authors performed 18F-PI-2620 in vitro autoradiography in four PSP cases that were independent of the in vivo cohort. The in vitro analyses revealed a blockable 18F-PI-2620 binding in the basal ganglia and the frontal cortex that was colocalized to AT8-positive aggregated tau. In vivo, patients with PSP showed significantly higher tracer binding in the pallidum and the nucleus subthalamicus than healthy controls. Binding in cortical regions could not differentiate between PSP and controls and there was no correlation between symptom severity and 18F-PI-2620 binding. The analyses at the single patient level revealed a sensitivity of 80% for PSP-RS and 55% for PSP-non-RS at a specificity of 83%. It should be elucidated that a cortical 18F-PI-2620 binding could be assessed in vitro but astonishingly there were no differences in cortical binding between PSP and controls in vivo. An autoradiographic study shed further light on the binding properties of 18F-PI-2620 to PSP tau in vitro [175]. Therefore, the authors compared the autoradiographic binding of 18F-PI-2620 in PSP, AD, and healthy controls for formalin-fixed paraffin-embedded (FFPE) and frozen tissue with immunohistochemical AT8 staining. Interestingly in FFPE samples, autoradiographic and immunohistochemical tau load correlated significantly in PSP and AD, whereas in frozen tissue this correlation was only existing in AD. The binding ratios in PSP in FFPE were significantly lower than those in AD, the authors explain this with the lower tau load in PSP compared to AD and to different binding affinities in 4R- and 3R/4R-tauopathies [175]. In a study with micro autoradiography, 3H-PI-2620 signal (silver grains) correlated with immunohistochemical staining with an antibody for tau (MCH1) [176]. Therefore, Willroider et al. draw the conclusion that an FFPE-induced off-target source is unlikely and that the 4R-tau might be less preserved in frozen tissue [175]. In a neuropathological study examining AT8-staining in a patient with CBD who died two weeks after a PET scan with 18F-PI-2620, the PET signal did not correlate with the AT8-staining [177]. Figure 4 (top row) shows PET imaging of a patient with probable PSP. The prior FDG-PET was not distinct for PSP. The additional tau PET with 18F-PI2620 revealed tau depositions in the basal ganglia. 

Another study shed further light on the role of tau imaging in CBS. Palleis et al. examined patients with CBS with 18F-PI-2620 and included amyloid-positive (3R/4R), confirmed by amyloid PET or CSF, as well as amyloid-negative cases (4R) [178]. Contrary to the prior study about patients with PSP [174], they could differentiate patients from controls also in cortical regions, albeit the signal of 18F-PI-2620 was higher in the amyloid positive group, leading to a sensitivity of 91% for 3R/4R-CBS and a sensitivity of 65% in 4R-CBS (CBD).

In this context, Song et al. examined whether the binding characteristics of 18F-PI-2620 could differentiate between 3R/4R- and 4R-tauopathies by non-invasive reference tissue modeling [179]. Song et al. found that there was a higher delivery and efflux of 18F-PI-2620 in cortical but not in subcortical regions in 4R- as compared to 3R/4R-tauopathy. The DVR of 18F-PI-2620 was significantly higher in 3R/4R. The authors draw the conclusion that the tracer binds less stable to 4R-tau. A reason for this could be different configurations of tracer binding pockets of tau aggregates. In this context, it is worth mentioning a computational study by Zhou et al. They could demonstrate in silico that none of the second-generation tracers is selective to 4R tau. The authors conclude that computational modeling of tracer binding characteristics can help to develop new and highly selective 4R tracers [180]. Figure 4 (bottom row) shows PET imaging from a patient with CBS. The FDG-PET showed hypometabolism in the left precentral gyrus. The scan with 18F-PI2620 revealed tau depositions in this area.

An interesting further aspect of tau imaging might be the use of the perfusion phase in order to detect neuronal injury, which could save an additional prior FDG PET. This could lead to an earlier diagnosis, reduced exposure to radiation, lower costs, and more comfort for the patients. Therefore, Beyer et al. compared an acquisition of 0.5 to 2.5 min p.i. (summed early-phase), as well as the blood flow estimate (R1 image) using the simplified reference tissue model of 18F-PI-2620, with 30 to 50 min 18F-FDG in patients with tauopathies and/or parkinsonism. They found high regional correlations between 18F-PI-2620 perfusion and FDG metabolism. In addition, there was no difference between more or less experienced readers for interpretation and no clear advantage of the summed early-phase or R1 [181]. Similar results could be obtained for 18F-AV-1451 in the past [182]. Another study examined the feasibility of short imaging protocols for 18F-PI-2620 in PSP in order to reduce costs (scanning time) and increase patients’ comfort. Authors found out that a 0–40 min p.i. dynamic scan offers the best balance between medical accuracy and economic aims also including the possibility of therapy monitoring in the future [183]. 

Interestingly, Tezuka et al. compared 18F-PI-2620 and 18F-PM-PBB3 binding in a subject with PSP-CBS. Whereas 18F-PM-PBB3 indicated tau burden in the frontal and parietal cortex, 18F-PI-2620 did not. Therefore, the authors conclude that 18F-PI-2620 might not accurately reflect tau burden in PSP [177]. In vitro, PM-PBB3 staining and autoradiography revealed a strong accordance with silver impregnation and AT8-staining for coiled bodies, argyrophilic threads, tufted astrocytes, and astrocytic plaques in the striatum of PSP/CBD. In vivo, the sensitivity and specificity for separation between patients and healthy controls were 94% and 96% for the nucleus subthalamicus. In this region, there was a positive correlation between disease severity and SUVRs. Furthermore, with an increase in PSP rating scores, there was an expansion of 18F-PM-PBB3 binding to cortical regions [184]. Ling and colleagues could differentiate PSP patients from controls with a sensitivity of 85% and a specificity of 100% in a two-region-based approach with 18F-PM-PBB3. There was a positive correlation between PSP severity and SUVRs for several subcortical regions. The sensitivity and specificity were lower at 85% and 71% when PSP patients were compared to patients with α-synucleinopathies. Indeed, two patients with PD and three patients with MSA were also tau PET positive in the basal ganglia [185]. Reasons could be a wrong initial diagnosis, tau co-pathology, or an off-target binding, similar to the results reported for 18F-AV-1451 in MSA patients [186], since there was no tracer uptake in healthy controls. 

In a nutshell, imaging with second-generation tau tracer is a useful tool in the diagnosis of atypical parkinsonism. The most experience is available for 18F-PI-2620. The sensitivity for PSP-non-RS and CBD is lower than for the other entities. Nevertheless, there is still uncertainty regarding the binding properties of 18F-PI-2620 to 4R-tau in vivo and in vitro. So far, there are no clear explanations for the different affinity of 18F-PI-2620 to 4R- and 3R/4R-tau. Therefore, it is still possible that, albeit with the good differentiation between patients and controls, 18F-PI-2620 measures a parallel off-target associated pathology. 18F-PM-PBB3 seems to be an interesting alternative to 18F-PI-2620, but this tracer should be evaluated in more patients with atypical parkinsonism in order to shed further light on the uptake of the putamen of patients with MSA. The development of a tracer specific to 4R-tau, e.g., with computational modeling, would be an important step for the future. 

### 5.2. Imaging of α-Synuclein

The development of a tracer for α-synuclein is particularly challenging and, although much awaited, there is no tracer available to date. Indeed, a tracer for α-synuclein would not only help to differentiate idiopathic PD from atypical PD and different forms of atypical PD from each other but may also allow monitoring of target engagement in future therapies for α-synucleinopathies. Therefore, the first report in March 2022 of α-synuclein imaging in patients with MSA with 18F-ACI12589 developed by AC Immune sounded very promising. However, evidence is yet limited and α-synuclein deposition was only reported in the cerebellar peduncle and cerebellar white matter of two patients with MSA-C, raising the question of whether the tracer is more likely to bind to α-synuclein aggregates specific MSA. Up to now, it remains unclear whether the tracer is able to detect α-synuclein depositions in MSA-P (suspected in the putamen) as well as in idiopathic PD and DLB. Another interesting tracer is 11C-MODAG-001, which was developed from anle253b [187]. 11C-MODAG-001 has a high affinity to α-synuclein fibrils and only moderate affinity to tau amyloid. However, despite confirmed in vivo binding in fibril-inoculated rat striata, autoradiography did not show binding in human brain sections of DLB. Other promising approaches came from the off-target binding of tau tracers in α-synucleinopathies such as 11C-PBB3, leading to the development of PPB3 analogs such as C05-01. However, although binding in α-synucleinopathies was confirmed ex vivo, C05-01 also bound to amyloid and tau [188]. Another tracer, 18F-SF3, could also demonstrate binding to α-synuclein in a rat model; nevertheless, the tracer also showed high selectivity to tau and amyloid [189]. Other tracers such as dibenzylcinnamamide [190] or julolidine [191] derivates based on computational modeling are currently under development. However, why is it so difficult to develop a tracer for α-synuclein? As opposed to amyloid, α-synuclein depositions are located intracellularly and, as opposed to tau, the abundance of α-synuclein is very low. Furthermore, among the different types of α-synucleinopathies, α-synuclein deposits can be found in different cell types, i.e., neurons for PD, and oligodendrocytes for MSA and the fibrils themselves have different structures. Lastly, the structures of the fibrils vary between in vitro simulations and in animal models and measurements in the human brain. For detailed reviews about the development of PET tracers for α-synucleinopathies, see [192,193]. In the future, it remains to be seen whether a universal tracer can be developed that measures α-synuclein in vivo in humans. Although promising, more studies are needed to evaluate the potential of 18F-ACI-12589 for PET imaging.

## 6. New Perspectives for Tracer Development and Technical Advances

As exemplified by α-synuclein, the development of new, specific PET tracers for brain imaging can be challenging, due to multiple caveats [194]. First of all, the tracer has to cross the blood-brain barrier, which can happen via passive diffusion (adequate lipophilicity of the tracer) or an active transport. Though, even if a molecule is able to pass the blood-brain barrier, the tracer has to withstand efflux transporters shuttling the molecules back in the bloodstream. Once in the brain, specific binding is required, and the tracer needs to have high affinity (with low inter- and intra- subject variability) and selectivity (low off-target binding) for a biologically relevant target. Up to now, PET tracers were developed based on trial and error and on the screening of compounds under laboratory conditions. Therefore, the use of computational models to develop new PET tracers in silico may greatly accelerate this process and enhance its efficiency [195,196]. Indeed, computational modeling is able to predict target-binding properties and pharmacokinetic characteristics [195,197]. Considering target-binding properties, computational models can be subdivided into structure-based drug design (structure of the target is known), which may be determined using cryogenic electron microscopy (cryo-EM) [198], and ligand-based drug design (structure of the target is not available). As such, computational modeling of the affinity to different binding sites for the tau fibrils might be determinant to develop new tracers with improved selectivity, in particular for α-synuclein and 4R tau [180,195].

In addition, antibody-based PET tracers are currently developed, as they intrinsically benefit from high affinity and specificity and may be used for theragnostic, as carried out in oncology. However, contrary to conventional brain PET tracer design, based on small drug-like molecules able to cross the blood–brain barrier, antibody-based tracers come with new challenges due to their size and chemical properties. A strategy to overcome this problem is the use of a carrier-mediated transport, e.g., with the transferrin receptor [199]. This may prove useful for imaging α-synuclein in the brain, although targeting intracellular α-synuclein inclusions as present in PD and DLB remains particularly challenging [200]. All in all, computational modeling in silico combined with cryo-EM and antibody-based PET tracers represent groundbreaking advances, likely to extend the possibility of brain imaging for diagnosis, treatment, and research.

Furthermore, both acquisition and interpretation have benefited from recent technological advances for use in the clinical context and research. In particular, improved efficiency of SPECT cameras with higher count sensitivity and spatial and energy resolution is critical for both radiation safety using lower radioactive activity and shorter acquisition time, thereby reducing the patient risk and discomfort. In particular, novel large-field cadmium–zinc–telluride and the use of a 360° detector ring may combine high-quality images, and high-speed recording increased by twofold in clinical conditions [201,202]. Improved contrast may also be achieved using multi-pinhole (MPH) collimators for DAT-SPECT, close to striatal delineation with PET [203]. As demonstrated before, interpretation has greatly improved using normative atlas and validation of innovative segmentation methods [204], and may greatly benefit from the use of machine learning in particular for differential diagnosis and prediction of individual prognosis for precision-medicine and use in neuroprotection trials.

Furthermore, recent developments in spectroscopy and pharmacological MRI (using drug-based challenges likely to induce changes in hemodynamic signal) bear the promise to provide high-resolution maps with molecular specificity, although the nanomolar affinity of radiotracers is unlikely to be challenged.

## 7. Conclusions

Altogether, molecular imaging proves increasingly useful in PD and degenerative parkinsonian disorders, both to the physician in everyday clinical practice and to the researcher, with the advent of new radiotracers and analytical methods to investigate neurotransmission abnormalities and neurodegeneration. As such, a combination of clinical examination and imaging of specific targets as warranted by SPECT and PET radiotracers opens new possibilities for the diagnosis and individual prognosis for precision medicine. Moreover, molecular imaging has the potential to serve both as an enrichment and progression biomarker, in particular in prodromal patients, critical for efficient design in disease-modification trials [73,205].

Therefore, it is expected that multimodal imaging using nuclear and MR imaging allows to further disentangle the complex interplay between neurodegenerative [206] and compensatory plasticity [207], as recently exemplified for the serotonergic [26,88] and cholinergic [107,208] systems. This process will be facilitated by hybrid PET/MR scanners [209], dual-phase PET, new radiotracers, pharmacological challenges, and biased agonism providing functional selectivity [210], in addition to technological efforts to reduce scan time and radiation hazard. As such, molecular imaging is key to accelerating the development and evaluation of disease-modifying, neuroprotective therapeutic strategies.

## Data Availability

Not applicable.
